# Pretreatment MRI Radiomics Based Response Prediction Model in Locally Advanced Cervical Cancer

**DOI:** 10.3390/diagnostics11040631

**Published:** 2021-03-31

**Authors:** Benedetta Gui, Rosa Autorino, Maura Miccò, Alessia Nardangeli, Adele Pesce, Jacopo Lenkowicz, Davide Cusumano, Luca Russo, Salvatore Persiani, Luca Boldrini, Nicola Dinapoli, Gabriella Macchia, Giuseppina Sallustio, Maria Antonietta Gambacorta, Gabriella Ferrandina, Riccardo Manfredi, Vincenzo Valentini, Giovanni Scambia

**Affiliations:** 1 Fondazione Policlinico Universitario “Agostino Gemelli” IRCCS, 00168 Roma, Italy; benedetta.gui@policlinicogemelli.it (B.G.); rosa.autorino@policlinicogemelli.it (R.A.); maura.micco@policlinicogemelli.it (M.M.); jacopo.lenkowicz@guest.policlinicogemelli.it (J.L.); davide.cusumano@policlinicogemelli.it (D.C.); luca.boldrini@policlinicogemelli.it (L.B.); nicola.dinapoli@policlinicogemelli.it (N.D.); mariaantonietta.gambacorta@policlinicogemelli.it (M.A.G.); gabriella.ferrandina@gmail.com (G.F.); riccardo.manfredi@policlinicogemelli.it (R.M.); vincenzo.valentini@policlinicogemelli.it (V.V.); giovanni.scambia@policlinicogemelli.it (G.S.); 2 Istituto di Radiologia, Università Cattolica del Sacro Cuore, 00168 Roma, Italy; adele.pesce1987@gmail.com (A.P.); lucarusso.md@gmail.com (L.R.); salvatorepersiani@gmail.com (S.P.); 3 Gemelli Molise Hospital, Università Cattolica del Sacro Cuore, 86100 Campobasso, Italy; macchiagabriella@gmail.com (G.M.); giuseppina.sallustio@policlinicogemelli.it (G.S.)

**Keywords:** radiomics, MRI, cervical cancer, pathological response, prediction model

## Abstract

The aim of this study was to create a radiomics model for Locally Advanced Cervical Cancer (LACC) patients to predict pathological complete response (pCR) after neoadjuvant chemoradiotherapy (NACRT) analysing T2-weighted 1.5 T magnetic resonance imaging (MRI) acquired before treatment start. Patients with LACC and an International Federation of Gynecology and Obstetrics stage from IB2 to IVA at diagnosis were retrospectively enrolled for this study. All patients underwent NACRT, followed by radical surgery; pCR―assessed on surgical specimen―was defined as absence of any residual tumour. Finally, 1889 features were extracted from MR images; features showing statistical significance in predicting pCR at the univariate analysis were selected following an iterative method, which was ad-hoc developed for this study. Based on this method, 15 different classifiers were trained considering the most significant features selected. Model selection was carried out using the area under the receiver operating characteristic curve (AUC) as target metrics. One hundred eighty-three patients from two institutions were analysed. The model, showing the highest performance with an AUC of 0.80, was the random forest method initialised with default parameters. Radiomics appeared to be a reliable tool in pCR prediction for LACC patients undergoing NACRT, supporting the identification of patient risk groups, which paves treatment pathways tailored according to the predicted outcome.

## 1. Introduction

Cervical cancer (CC) represents the fourth leading cause of cancer death in women, with 311,000 deaths in 2018 worldwide [1]. Treatment depends mainly on the stage of the tumour at diagnosis, as assessed by the International Federation of Gynecology and Obstetrics (FIGO) 2009 staging system [2].

Locally advanced stages are usually treated with external beam radiotherapy in association with platinum-based chemotherapy (CRT) followed by brachytherapy boost [3]. Although survival rates for women with Locally Advanced Cervical Cancer (LACC) are improving, one in three women develop local and pelvic recurrences, which supports the hypothesis of residual disease presence after definitive chemoradiation therapy [4].

Data suggest a role of neoadjuvant chemoradiotherapy (NACRT) followed by radical hysterectomy to remove potential radio- and chemo-resistant neoplastic foci, which improves local control in nonresponding patients [5,6,7]. Furthermore, pathological complete response (pCR) was associated with higher disease-free and long-term survival [8,9,10]. Therefore, a pCR pretreatment prediction may have a significant impact on LACC patient management by identifying tailored approaches for patient subgroups to achieve better clinical results.

In this context, the application of medical imaging technologies has significantly developed during the last decade from a primarily qualitative analysis to a quantitative approach disclosing an immense amount of information. The revised FIGO staging permits the use of imaging and pathological findings, where accessible, to determine the stage. Regarding imaging technique the revised FIGO staging allows any of the imaging modalities depending on the accessible sources, such as ultrasound, CT, magnetic resonance imaging (MRI), positron emission tomography (PET), to give information regarding tumour dimension, nodal status and local and systemic extension [11]. MRI is the best imaging technique for the assessment of cervical lesions greater than 1 cm and it is recommended for initial imaging evaluation when tumour dimension is greater than 2 cm, showing to be effective for LACC staging and prognosis evaluation [12]. Different MRI sequences (T2WI, DW-MRI, DCE-MRI) have been assessed as noninvasive biomarkers of treatment response with variable and promising results [13,14,15]. In particular, T2-sequences and diffusion-weighted imaging (DWI) have been studied in this setting. However, the role of DWI and apparent diffusion coefficient (ADC), to monitor early treatment response in patients affected by LACC undergoing CRT, is still controversial in literature [14,16]. Moreover, the future direction in term of prognostic noninvasive biomarkers should include the combination of different imaging technique such us US, CT, MRI and PET integrated with clinical and histologic data.

Radiomics is to date a rapidly expanding field of clinical research, which gives the possibility to quantify intratumoural heterogeneity in a high throughput and noninvasive way, and offers the chance to individuate risk groups for single patients to allow tailored treatment according to the predicted outcome [17,18,19]. Indeed, tumour heterogeneity showed significant correlations with radiomics in a variety of cancer patients, including cervical cancer [20,21,22].

Some preliminary studies reporting CC pretreatment experiences used MRI images to characterise cervical lesions [13,14], to predict local response and to assess biological tumour heterogeneity [23]. However, no studies have correlated pretreatment MRI radiomics and histopathology in LACC patients treated with NACRT followed by radical hysterectomy.

The aim of this study was to investigate the potential role of MRI radiomics, used for staging, to predict pCR following NACRT in LACC patients with different FIGO stages.

## 2. Materials and Methods

### 2.1. Patient Enrolment and Image Acquisition Protocol

Patients affected by LACC, with FIGO stage from IB2 to IVA, treated in two different institutions, were considered for this retrospective analysis.

The cohort of institution A consisted of patients treated between 2007 and 2014; the cohort of institution B included cases treated from 2005 to 2013. Inclusion criteria were histological confirmed invasive carcinoma of the cervix, FIGO stage from IB2 to IVA, and absence of distant metastasis (cM0). Patients younger than 18 years, without pretreatment MRI, treated with palliative intent or those who did not undergo surgery for histological confirmation of response, were excluded from the analysis. Table 1 summarises a complete description of the cohorts investigated in this study.

In both centres, a 1.5 T MR machine (GE Signa Exite, Little Chalfont, UK) was used for imaging.

### 2.2. Treatment Workflow and Response Assessment

All patients underwent NACRT. Radiotherapy volumes were delineated according to consensus guidelines [24]. Concurrent chemotherapy was administrated with cisplatin alone or cisplatin plus 5-fluorouracil [25,26]. Radiotherapy consisted of 50.6 Gy administration to the primary tumour (PTV1) and 45 Gy to nodal drainage (PTV2) or 45 Gy to PTV1 and PTV2, according to the clinical disease stage. Restaging was performed 4–6 weeks after NACRT completion with MRI and PET-CT. All patients underwent radical hysterectomy plus pelvic lymphadenectomy within 6–8 weeks from NACRT completion. Pathological response to treatment was evaluated on surgical specimens. Complete pathological response (pCR) was defined as absence of any residual tumour after treatment at any site; microscopic response (pR1) as persistent tumour foci of maximum dimension inferior to 3 mm; macroscopic response (pR2) as persistent tumour foci with maximum dimension exceeding 3 mm [27].

### 2.3. Image Analysis

MRI for staging was acquired according to local institutional diagnostic protocols without injecting contrast agents. Acquisition parameters adopted in the MR protocol are reported in Table 2.

The gross tumour volume (GTV) was contoured on the axial T2-weighted MR images. GTV was manually contoured in consensus by two radiologists, experts in gynaecological imaging, using a radiotherapy treatment planning system (TPS) (Eclipse, Varian Medical Systems, Palo Alto, CA, USA).

The segmented images were then processed using MODDICOM, an R library designed to perform radiomic analysis. Before starting the radiomic analysis, image resolution was homogenised and all MR images were resampled to a value of spatial planar resolution equal to 0.548 × 0.548 mm^2^ [28,29].

### 2.4. Feature Extraction

Once resampled, MR images were processed using the Laplacian of Gaussian (LoG) or the intensity based (IB) image filter. The LOG filter was applied varying the σ parameter, which is a measure of filter width, in the range 0–4.2 mm with steps of 0.35 mm (13 steps in total). Regarding the IB filter, a preliminary normalisation of the pixel intensities inside the region of interest (ROI) was performed, using the first and 99th percentile of ROI histogram grey levels as extremes. Pixel clusters were then identified considering two threshold levels (lower and upper level), defined as percentages of the maximum intensity level [30].

The IB filter was applied considering all the possible combinations of thresholds for levels ranging from 0% to 100% by 10% steps (for 55 combinations).

The feature extraction was then performed on the filtered images considering the GTV as ROI: in particular, first-order features were calculated on the images processed with the LOG filter, while fractal, textural and morphological features were calculated considering the MR images processed using the IB filter.

Considering the application of all the filters used, a total of 1889 radiomic features were extracted. The computer code used for image analysis can be found at https://bitbucket.org/kboadmin/lacc_radiomics/src/master/ (accessed date: 24 February 2020), while the comprehensive list of the features calculated is reported in the IBSI initiative [31].

### 2.5. Statistical Analysis

The two patient cohorts were merged in a unique training set: feature selection and model training were carried out following an iterative method, ad-hoc developed for this study.

This process consisted in a first step of feature selection and in a following step of classifier selection.

Concerning the feature selection step, an eight-fold cross validation was performed: the merged dataset was randomly divided in eight folders and eight different datasets were analysed, considering each time seven of the eight folders defined.

For each combination of the seven folders selected, Wilcoxon Mann–Whitney (WMW) test (or Student’s t-test depending on normality of the data distribution) was computed to investigate the ability of each radiomic feature in predicting the defined outcome at the univariate analysis.

Features, resulting at least five times on the eight folder combinations significant (*p* < 0.05), were selected for correlation analysis, which was computed calculating the Pearson correlation coefficient (PCC). The final set of significant features was composed by features showing a correlation value inferior to 0.6, to remove features with moderate mutual correlation [32].

Regarding the model selection, 15 different classifiers were trained considering significant features selected by previous analysis.

Table 3 reports the extended names and the caret method of each of the tested models.

**Figure 1:**
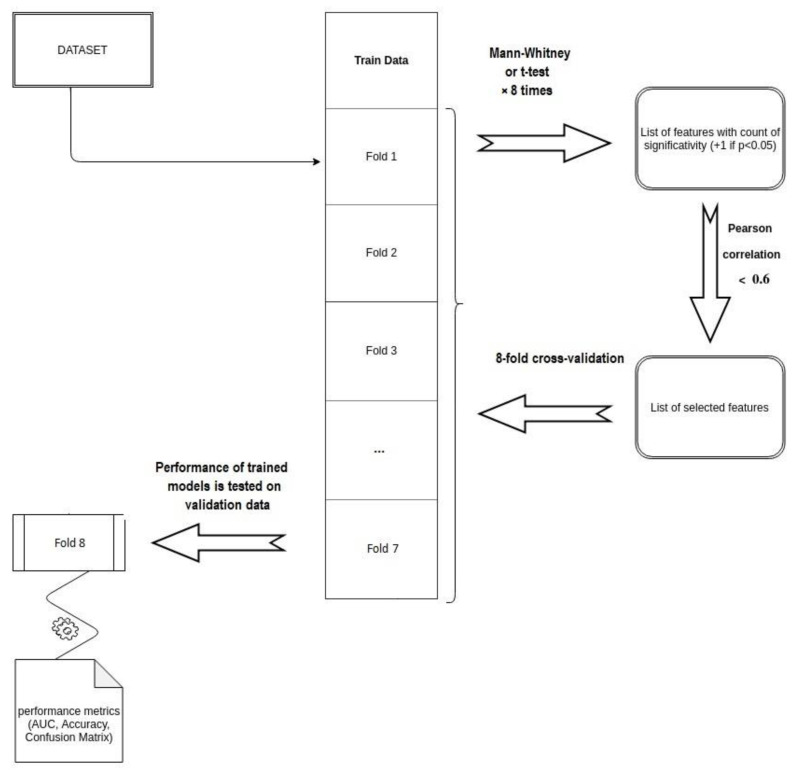
Scheme of the workflow used for features and model selection.

Even in this case, the merged dataset was partitioned in eight folders and each classifier was trained on seven of the eight folders considered, using the remaining folder as validation set. For each classifier, eight receiver operating characteristic (ROC) curves were calculated, one for each unique combination of folders. The area under these ROC curves (AUC) was considered as metric to identify the best model. The model selection process was repeated three times, modifying the random selection of the cases in the eight folders. At the end of the three iterations, 24 ROC curves were analysed for each classifier, and the mean AUC value with the corresponding standard deviation was calculated. The model showing the highest mean AUC value on the three iterations of the cross-validation analysis was considered as the best classifier [33]. The best classifier was then trained on the whole dataset to obtain the final predictive model. The scheme of the entire workflow adopted for the features and model selection is reported in Figure 1.

The performance of the final predictive model was quantified in terms of sensitivity, specificity and accuracy at the optimal discriminative threshold, which was identified through the Youden Index calculation, as reported in similar experiences [34,35].

All analyses described were performed using the caret package of the R software (version 3.4.3, 2017; www.r-project.org (accessed on 25 February 2021)).

## 3. Results

In total, 183 patients were included in the analysis. Out of 156 patients of cohort A, 66 (42%) pCR, 45 (29%) pR1 and 45 (29%) pR2 were observed. In cohort B (*n* = 27), we observed 8 (30%) patients with pCR, 9 (33%) with pR1 and 10 (37%) with pR2. The feature selection process identified 19 radiomic features significant at the univariate analysis.

Starting from the identified significant features, a total of 15 predictive models were calculated: Table 4 reports the mean AUC values obtained on the 24 iterations performed during model selection for 15 classifiers considered, together with the standard deviation (SD) value, which was considered as measurement of the variation in predictive performance of the elaborated models.

The AUC of the ROC curves calculated on the 24 iterations performed for the 15 classifiers considered in the work are provided as Supplementary Materials.

**Figure 2:**
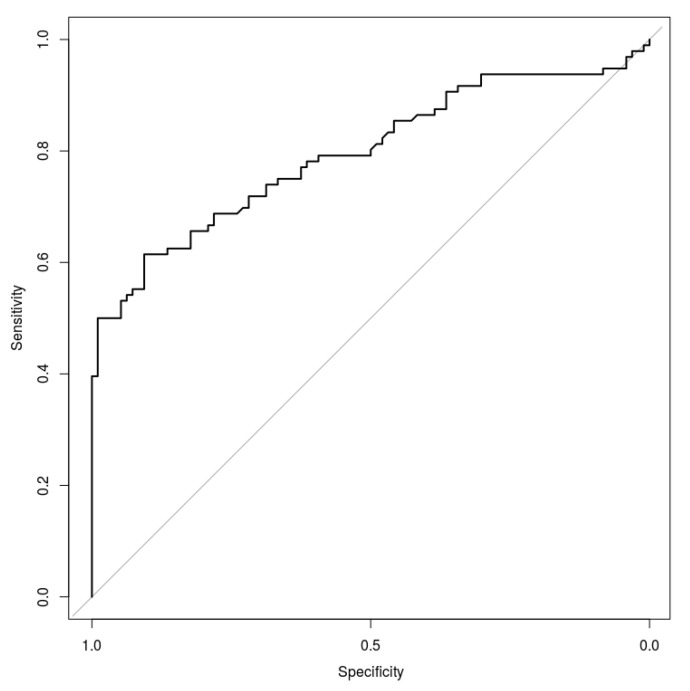
Receiver operating characteristic (ROC) curve obtained considering the random forest model initialised with default parameters.

The model showing the highest performance was the random forest (RF_DEF), which was initialised with the following default parameters: number of trees (ntree) equal to 500, and number of variables randomly sampled as candidates for each split (mtry) set equal to 4, which corresponds to the square root of variable numbers for classification. The final ROC curve model trained on the whole dataset is reported in Figure 2.

The RF_DEF model showed a mean AUC of 0.80 ± 0.8. The best cut off threshold was equal to 0.69 which corresponds to a Youden Index of 0.486.

At the best cut-off threshold, the present model reports a specificity of 98.4% and a sensitivity of 50.2%, with an overall accuracy of 74.5%.

**Figure 3:**
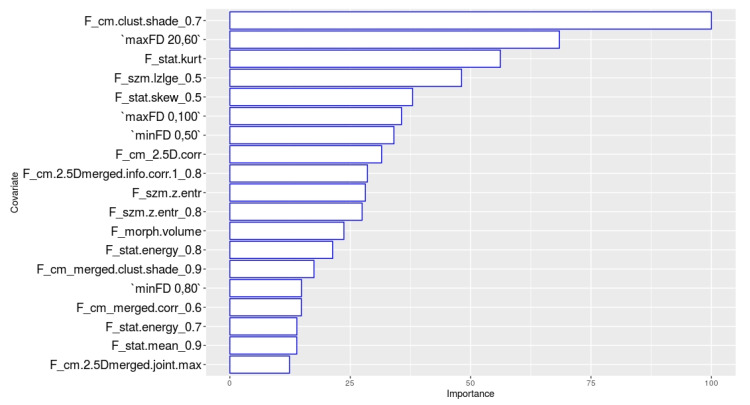
Importance of the parameters included in the model.

The importance of the single variables included in the random forest model is reported in Figure 3.

The most important feature of the model is the cluster shade calculated on the grey level co-occurrence matrix, after the application of the LOG filter on the raw images with a σ value of 0.7.

The other two features showing an importance higher than 50% are the maximum fractal dimension calculated on the subpopulation from 20% to 60% of the maximum pixel value and the kurtosis calculated on the raw MR images.

## 4. Discussion

Although NACRT followed by surgery is not a care standard for LACC patients in many centres, this approach achieved encouraging results with high rates of pathologically assessed complete response and local control rates with acceptable toxicity [8,9].

In this context, a tool to identify specific subgroups of patients who can benefit from a given treatment is necessary to achieve a fully personalised clinical approach. Several studies aimed to predict and monitor treatment response and clinical outcome analysing functional imaging like MRI or 18-FDG PET-TC in CC patients [23,36,37,38]. Literature suggests that metabolic response in post-therapy PET-CT correlates with failure patterns [36] and can predict OS in patients treated with CRT [37]. Some studies focused on temporal changes in tumour heterogeneity patterns on functional imaging such as dynamic contrast enhanced MRI, DWI [23] and FDG PET-CT [38] performed before, during and after CRT course to correlate imaging with treatment response outcomes and to define prognostic factors. In particular, tumour volume and ADC value were the most important prognostic factors [12,13,14,22]. However, the role of functional imaging is still under investigation in literature.

Promising evidence supports the use of delta radiomics assessment in predicting outcomes (e.g., response to therapies) for treatment personalisation [23,39]. To the best of our knowledge, this study represents the first radiomics based pCR prediction model on pretreatment staging MRI in patients affected by locally advanced cervical cancer undergoing NACRT. The choice of pCR as outcome parameter represents the strength of this study; in fact, surgery following CRT allows verifying the pathological response to the treatment. The majority of studies on response prediction in CC is focused on early treatment response assessment during or soon after NACRT.

The proposed model allows to predict the probability of achieving pCR using pretreatment imaging to allow clinicians the possibility to better plan the entire treatment using routinely acquired imaging for disease staging.

Clinicians can also consider new protocols of dose escalation or dose-de-intensification based on single patient’s predicted outcomes, in order to ensure a response to treatment. This approach could significantly save resources in the management of this patient population optimising treatment workflows based on outcome prediction.

The technical robustness of the model selection procedures and the encouraging AUC values (0.80) of the random forest method proposed represent an innovative tool for clinical decision support and oncological treatment personalisation. However, one of the main weaknesses of this predictive model is the low degree of direct interpretability of the individual features extracted from the MR images; the proposed method acts as a black-box, limiting the clinical interpretability of the key parameters on which this model is based. The poor interpretability of the model elaborated represents one of the main limitations of the modern techniques of image analysis, such as machine learning and deep learning: several experiences have already highlighted these limitations and new research fields are currently aiming to explain the artificial cognitive processes of these techniques [40,41].

Another limitation is represented by the lack of an external validation dataset, which is partially overcome by means of cross-validation: further studies aiming to externally validate this model are recommended in the future.

The present study was performed considering only T2-w MR images, as it was the only imaging modality available for all patients included in the study. Other MR imaging modalities, such as DWI or dynamic contrast enhanced sequences, were object of radiomic analysis in some experiences [20,23,42,43], and not considered in other studies based only on T2-w MR image analysis [42,43,44].

Future developments of this innovative approach can therefore take into account external validation cohorts with images acquired on scanners provided by different vendors, as already performed for other tumour sites like rectal cancer [45]. Moreover, the inclusion of DWI ADC-maps segmentation should be evaluated in future studies in order to find other radiomic features useful for pCR prediction [46,47].

These innovative imaging approaches could play a fundamental role in hybrid magnetic resonance guided radiotherapy (MRgRT) treatments, optimising radiotherapy planning of radiomics’ analysis output through the quantification of feature changes throughout treatment. Taken this into consideration, new dose delivery and targeting paradigms could be proposed and better treatment outcomes may be achieved, as already demonstrated in rectal and cervical cancer [48,49].

In conclusion, this radiomics based prediction pCR model can be useful to guide clinicians in their decision-making process, tailoring treatment according to response prediction in the frame of fully personalised clinical management of cervical cancer care.

## Figures and Tables

**Table 1:** Patient characteristics.

	Institution A (156 pts)	Institution B (27 pts)
Age (Mean)	22–76 (50.2)	28–79 (54.2)
Histology		
Squamous cell carcinoma	142 (91%)	23 (85.2%)
Glassy cell squamous carcinoma	0	1 (3.7%)
Clear cell adeno-squamous carcinoma	1 (0.7%)	0
Adenocarcinoma	12 (7.6%)	2 (7.4%)
Adeno-squamous	1 (0.7%)	1 (3.7%)
FIGO Stage		
IB2	6 (3.8%)	2 (7.4%)
IIA	9 (5.8%)	2 (7.4%)
IIB	119 (76.3%)	21 (77.8%)
IIIA	6 (3.8%)	2 (7.4%)
IIIB	13 (8.4%)	0
IVA	3 (1.9%)	0
Nodal status		
N0	75 (48.1%)	17 (63%)
N1	81 (51.9%)	10 (37%)
Pathological Response		
pR0	66 (42.4%)	8 (29.7%)
pR1	45 (28.8%)	9 (33.3%)
pR2	45 (28.8%)	10 (37%)

pR0: absence of any residual tumour after treatment at any site; pR1: microscopic response as persistent tumour foci of maximum dimension inferior to 3 mm; pR2: macroscopic response as persistent tumour foci with maximum dimension exceeding 3 mm.

**Table 2:** Magnetic resonance imaging (MRI) acquisition parameters used in the MR clinical protocol adopted for axial (AX), sagittal (SAG) and coronal (COR) acquisitions.

	AX T1-W	AX T2-W	SAG T2-W	AX OBLIQUE T2-W (Perpendicular to the Long Axis of the Cervix)	COR OBLIQUE T2-W (Parallel to the Long Axis of the Cervix)	AX ABDOMINAL T2-W	AX OBLIQUE DWI (= Ax Oblique T2-w)
Sequence	FSE	FRFSE	FRFSE	FRFSE	FRFSE	FRFSE- XL	EPI
Echo time (ms)	16	85	85	85	85	84	Minimum
NEX	2	2	2	4	4	1	6
Repetition time (ms), TR	470	4500	4500	4500	4500	1850	5425
No. of sections	30	30	26	16	16	48	30
Receiver bandwidth (kHz)	31.25	31.25	41.67	41.67	41.67	41.67	
Echo train length	3	26	15	26	26	17	
Field of view (mm), FOV	24	24	24	22	24	46	28
Section thickness (mm)	4	4	4	3	4	5	4
Section spacing (mm)	0.5	0.5	0.4	0.5	0.5	1	0.5
Matrix size	448 × 288	384 × 256	384 × 256	384 × 256	384 × 256	256 × 256	128 × 128
*b* Value (s/mm^2^)	---	---	---	---	---	---	800
Phase direction	A/P	A/P	S/I	UNSWAP	UNSWAP	R/L	R/L

**Table 3:** Models’ legend, extended name and method used to calculate the classifier using the caret package of the R statistical software.

Model	Extended Name	Caret Method
C5TREE	Decision tree	C5.0Tree
DT	Decision tree	C5.0
HDDA	High dimensional discriminant analysis	hda
KNN	K-nearest neighbours	kknn
LOGREG	Logistic regression	glm
NB	Naive Bayes	nb
NN	Neural network	nn
PAM	Nearest shrunken centroids	pam
PDA	Penalised discriminant analysis	pda
PLS	Partial least square	pls
RF_DEF	Random forest	rf. Default parameters
RF_GRID	Random forest	rf. Grid search
RF_RAND	Random forest	rf. Random search
SDA	Shrinkage discriminant analysis	sda
SVM	Support vector machine	svmPoly. Polynomial Kernel

**Table 4:** Mean and standard deviation (SD) values obtained in terms of area under the receiver operating characteristic curve (AUC) on the 24 iterations performed for model selection.

Model	Mean AUC	SD (AUC)
RF_DEF	0.80	0.08
RF_GRID	0.79	0.11
RF_RAND	0.79	0.07
NN	0.73	0.11
SVM	0.69	0.11
PLS	0.68	0.10
PDA	0.68	0.11
DT	0.67	0.13
NB	0.67	0.09
SDA	067	0.10
KNN	0.66	0.09
LOGREG	0.66	0.12
PAM	0.64	0.12
HDDA	0.63	0.09
C5TREE	0.63	0.11

## Data Availability

The raw data supporting the conclusions of this article will be made available by the authors upon request, without undue reservation.

## Supplementary Materials

The following are available online at https://www.mdpi.com/article/10.3390/diagnostics11040631/s1, Table S1: AUC of the ROC curves calculated on the 24 iterations performed for the 15 classifiers considered in the work.

## Abbreviations

LACC	Locally Advanced Cervical Cancer;
pCR	pathological complete response;
NACRT	neoadjuvant chemoradiotherapy;
MRI	magnetic resonance imaging;
AUC	area under the curve;
CC	cervical cancer;
FIGO	International Federation of Gynecology and Obstetrics;
CRT	chemoradiotherapy;
DWI	diffusion-weighted imaging;
ADC	apparent diffusion coefficient;
PTV	planned target volume;
GTV	gross tumour volume;
TPS	treatment planning systems;
LoG	Laplacian of Gaussian;
IB	intensity based;
ROI	region of interest;
WMW	Wilcoxon Mann–Whitney;
PCC	Pearson correlation coefficient;
ROC	receiver operating characteristic;
SD	standard deviation;
RF_DEF	random forest model;
Ntree	number of trees;
Mtry	number of variables randomly sampled as candidates for each split;
OS	overall survival;
MRgRT	magnetic resonance guided radiotherapy.
