# Supplementary material for: Pretreatment MRI Radiomics Based Response Prediction Model in Locally Advanced Cervical Cancer
Source: Diagnostics (Basel). 2021 Mar 31;11(4):631. doi: 10.3390/diagnostics11040631 (PMC8066099; doi:10.3390/diagnostics11040631)
Supplement: Supplementary file 1 [file diagnostics-11-00631-s001.pdf]

Supplementary Materials

Table S1 – AUC of the ROC curves calculated on the 24 iterations performed for the 15 classifiers considered in the work.

Iterations	*DT*	*LOGREG*	*C5TREE*	*HDDA*	*KNN*	*PAM*	*PDA*	*PLS*	*SDA*	*NB*	*NN*	*RF_DEF*	*RF_GRID*	*RF_RAND*	*SVM*
1	0.87	0.89	0.84	0.60	0.79	0.47	0.69	0.83	0.72	0.88	0.83	0.70	0.81	0.75	0.85
2	0.67	0.71	0.55	0.60	0.83	0.61	0.72	0.80	0.75	0.60	0.90	0.67	0.76	0.80	0.69
3	0.60	0.57	0.70	0.58	0.75	0.62	0.69	0.74	0.44	0.60	0.71	0.81	0.89	0.90	0.81
4	0.59	0.65	0.54	0.57	0.71	0.88	0.79	0.73	0.48	0.62	0.81	0.92	0.78	0.88	0.74
5	0.66	0.55	0.58	0.57	0.63	0.58	0.53	0.65	0.63	0.56	0.72	0.75	0.88	0.84	0.51
6	0.53	0.79	0.71	0.68	0.67	0.72	0.60	0.60	0.67	0.57	0.69	1.00	0.85	0.73	0.68
7	0.90	0.47	0.72	0.74	0.58	0.65	0.83	0.49	0.85	0.74	0.86	0.81	0.83	0.83	0.58
8	0.63	0.79	0.50	0.46	0.77	0.58	0.58	0.45	0.60	0.67	0.82	0.63	0.75	0.77	0.78
9	0.78	0.87	0.42	0.64	0.58	0.71	0.52	0.68	0.79	0.75	0.69	0.99	0.92	0.80	0.83
10	0.70	0.58	0.52	0.72	0.58	0.61	0.61	0.65	0.71	0.77	0.70	0.69	0.77	0.83	0.69
11	0.43	0.53	0.66	0.65	0.50	0.51	0.70	0.72	0.60	0.74	0.81	0.75	0.85	0.75	0.53
12	0.64	0.61	0.53	0.59	0.67	0.51	0.64	0.60	0.66	0.73	0.62	0.78	0.74	0.83	0.72
13	0.85	0.63	0.66	0.80	0.58	0.65	0.71	0.65	0.76	0.78	0.72	0.81	0.71	0.78	0.80
14	0.80	0.53	0.57	0.58	0.67	0.58	0.58	0.79	0.65	0.56	0.69	0.90	0.58	0.69	0.56
15	0.61	0.65	0.76	0.63	0.58	0.83	0.90	0.84	0.54	0.48	0.55	0.63	0.83	0.67	0.60
16	0.75	0.81	0.64	0.86	0.63	0.60	0.89	0.72	0.75	0.64	0.81	0.78	0.81	0.80	0.70
17	0.85	0.54	0.57	0.53	0.63	0.83	0.70	0.66	0.63	0.70	0.81	0.62	0.90	0.68	0.62
18	0.73	0.60	0.57	0.73	0.58	0.53	0.56	0.53	0.67	0.61	0.72	0.88	0.76	0.70	0.68
19	0.53	0.78	0.75	0.67	0.75	0.75	0.53	0.59	0.70	0.69	0.73	0.75	0.70	0.74	0.85
20	0.69	0.59	0.60	0.56	0.63	0.72	0.72	0.70	0.67	0.60	0.67	0.88	0.80	0.76	0.53
21	0.69	0.58	0.65	0.53	0.68	0.60	0.81	0.72	0.83	0.75	0.40	0.90	0.94	0.83	0.63
22	0.66	0.77	0.82	0.58	0.81	0.67	0.61	0.73	0.73	0.72	0.81	0.70	0.82	0.90	0.70
23	0.37	0.72	0.48	0.58	0.58	0.65	0.70	0.71	0.53	0.68	0.82	0.74	0.69	0.83	0.74
24	0.66	0.59	0.70	0.75	0.79	0.40	0.63	0.66	0.68	0.60	0.71	0.86	0.88	0.83	0.83
Mean	**0.67**	**0.66**	**0.63**	**0.63**	**0.66**	**0.64**	**0.68**	**0.68**	**0.67**	**0.67**	**0.73**	**0.79**	**0.80**	**0.79**	**0.69**
St Deviation	**0.13**	**0.12**	**0.11**	**0.09**	**0.09**	**0.12**	**0.11**	**0.10**	**0.10**	**0.09**	**0.11**	**0.11**	**0.08**	**0.07**	**0.11**
